# *Bifidobacterium bifidum* Enhances the Intestinal Epithelial Tight Junction Barrier and Protects against Intestinal Inflammation by Targeting the Toll-like Receptor-2 Pathway in an NF-κB-Independent Manner

**DOI:** 10.3390/ijms22158070

**Published:** 2021-07-28

**Authors:** Rana Al-Sadi, Viszwapriya Dharmaprakash, Prashant Nighot, Shuhong Guo, Meghali Nighot, Toan Do, Thomas Y. Ma

**Affiliations:** Department of Medicine, Penn State College of Medicine, Hershey Medical Center, Penn State University, Hershey, PA 17033, USA; ralsadi@pennstatehealth.psu.edu (R.A.-S.); vdharmaprakash@pennstatehealth.psu.edu (V.D.); pnighot@pennstatehealth.psu.edu (P.N.); sguo2018@uw.edu (S.G.); mnighot@pennstatehealth.psu.edu (M.N.); doto@ohsu.edu (T.D.)

**Keywords:** *Bifidobacterium*, intestinal barrier, toll-like receptor-2

## Abstract

Defective intestinal tight junction (TJ) barrier is a hallmark in the pathogenesis of inflammatory bowel disease (IBD). To date, there are no effective therapies that specifically target the intestinal TJ barrier. Among the various probiotic bacteria, *Bifidobacterium*, is one of the most widely studied to have beneficial effects on the intestinal TJ barrier. The main purpose of this study was to identify *Bifidobacterium* species that cause a sustained enhancement in the intestinal epithelial TJ barrier and can be used therapeutically to target the intestinal TJ barrier and to protect against or treat intestinal inflammation. Our results showed that *Bifidobacterium bifidum* caused a marked, sustained enhancement in the intestinal TJ barrier in Caco-2 monolayers. The *Bifidobacterium bifidum* effect on TJ barrier was strain-specific, and only the strain designated as BB1 caused a maximal enhancement in TJ barrier function. The mechanism of BB1 enhancement of intestinal TJ barrier required live bacterial cell/enterocyte interaction and was mediated by the BB1 attachment to Toll-like receptor-2 (TLR-2) at the apical membrane surface. The BB1 enhancement of the intestinal epithelial TJ barrier function was mediated by the activation of the p38 kinase pathway, but not the NF-κB signaling pathway. Moreover, the BB1 caused a marked enhancement in mouse intestinal TJ barrier in a TLR-2-dependent manner and protected against dextran sodium sulfate (DSS)-induced increase in mouse colonic permeability, and treated the DSS-induced colitis in a TJ barrier-dependent manner. These studies show that probiotic bacteria BB1 causes a strain-specific enhancement of the intestinal TJ barrier through a novel mechanism involving BB1 attachment to the enterocyte TLR-2 receptor complex and activation of p38 kinase pathway.

## 1. Introduction

Intestinal epithelial tight junction (TJ) barrier dysfunction has been implicated as an important pathogenic factor of various inflammatory conditions of the gut, including inflammatory bowel disease (IBD), necrotizing enterocolitis (NEC), and celiac disease [1,2,3,4]. Clinical studies in IBD patients have shown that a persistent increase in intestinal permeability following clinical remission is predictive of poor clinical outcome and an early recurrence of the disease [5,6], while normalization of intestinal permeability correlated with a sustained long-term clinical remission. Transmission electron microscopy and freeze-fracture analysis of intestinal tissues has also shown that patients with IBD have separation of intercellular spaces between intestinal epithelial cells and decreased number of TJ strands [7,8], consistent with the presence of defective TJ barrier and increased paracellular permeation of luminal contents. There has been much interest in the role of the intestinal microbiome on IBD and the use of probiotics to modulate and treat intestinal inflammation [9,10]. The beneficial effects of probiotics in the treatment of IBD have been relatively modest and mostly limited to pouchitis [11]. There is an important unmet need and a gap in scientific knowledge related to the use of probiotic bacterial species that can target and preserve the intestinal epithelial TJ barrier function and also prevent and treat intestinal inflammation.

*Bifidobacterium* is an important genus consisting of probiotic bacterial species that predominate intestinal flora of breastfed infants and are associated with improved intestinal barrier and infant health [9,12,13,14,15]. Among the various probiotic bacteria in the infant’s gut reported to date, *Bifidobacterium*, is one of the most widely studied and utilized probiotic bacteria and are the first to colonize the human intestine, a phenomenon driven by the bifidogenic activities of certain mother milk-derived oligosaccharides [16,17]. Studies in both human and animal models have suggested that *Bifidobacterium* protects the intestinal barrier, and prophylactic administration of *Bifidobacterium* has been associated with beneficial effects in intestinal inflammation [18,19,20,21,22,23]. Although many studies have supported the health-promoting activities of *Bifidobacteria* species, it remains unclear as to which *Bifidobacterium* species produces an enhancement in the intestinal TJ barrier and which may be used therapeutically to target the intestinal TJ barrier and to protect against the development of intestinal inflammation. The main purpose of this study was to identify *Bifidobacterium* species that causes a sustained enhancement in the intestinal epithelial TJ barrier, and which may be used therapeutically to target the intestinal TJ barrier and to protect against or treat intestinal inflammation.

We examined the effects of various *Bifidobacterium* species on intestinal epithelial TJ barrier function. We found that one species, in particular, *Bifidobacterium bifidum* (BB), caused a marked increase in intestinal TJ barrier function in cell culture and in a live mouse. Our studies also revealed that BB caused a strain-specific tightening of the intestinal TJ barrier, which was mediated in part by BB attachment to Toll-like receptor-2 (TLR-2) complex on the apical membrane surface of the enterocyte. These studies also showed that BB protected against dextran sodium sulfate (DSS)-induced colitis by targeting the intestinal TJ barrier in mice.

## 2. Results

### 2.1. Effect of Bifidobacterium Species on Caco-2 Intestinal Epithelial TJ Barrier Function

**Figure 1:**
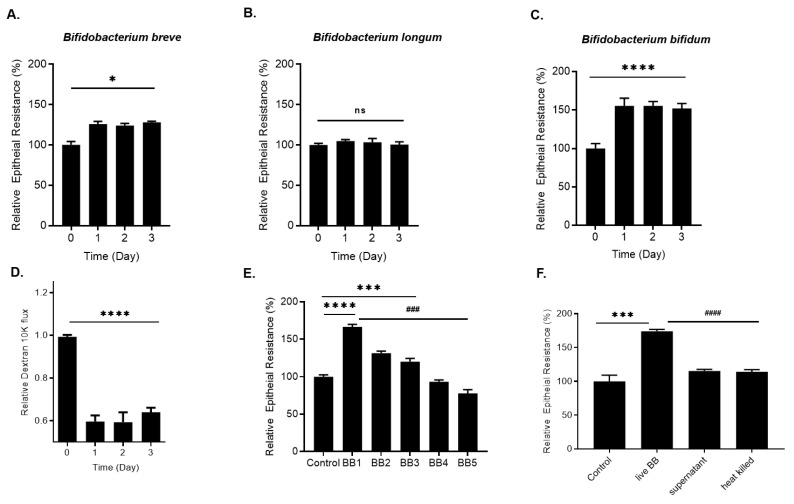
Effect of *Bifidobacterium* species and strains (1 × 10^8^ CFU/mL) on filter-grown Caco-2 TJ permeability. (**A**) Time-course effect of *Bifidobacterium breve*, (**B**) *Bifidobacterium longum, and* (**C**) *Bifidobacterium bifidum* on Caco-2 TER; * *p* < 0.05 vs. control; **** *p* < 0.0001 vs. control. (**D**) Time-course effect of *Bifidobacterium bifidum* (BB) on Caco-2 mucosal-to-serosal flux of paracellualr marker dextran 10 kDa; **** *p* < 0.0001 vs. control. (**E**) Strain BB1 caused a 50–80% increase in Caco-2 TER, BB2 and BB3 strains caused a ~25% increase in Caco-2 TER, but BB4 and BB5 did not enhance Caco-2 TER over the 3-day experimental period; **** *p* < 0.0001 vs. control; *** *p* < 0.001 vs. control. ### *p* < 0.001 vs. BB1. (**F**) Live BB1, but neither BB1 bacterial supernatant nor heat-killed BB1 caused an increase in filter-grown Caco-2 TER; *** *p* < 0.001 vs. control; #### *p* < 0.0001 vs. live BB1; ns: not significant.

We examined the effects of *Bifidobacterium* species on intestinal epithelial TJ barrier function by measuring trans-epithelial resistance (TER) in filter-grown Caco-2 monolayers. Three commonly used *Bifidobacterium* species, *Bifidobacterium bifidum, Bifidobacterium breve, and Bifidobacterium longum* (1 × 10^8^ CFU/mL), were applied to the apical compartment of filter-grown Caco-2 monolayers for up to a 3-day experimental period. The bacterial concentration of 1 × 10^8^ CFU/mL was maintained throughout the entire experimental period by daily rinsing off of the probiotic bacteria from the apical compartment and recharging the apical compartment with 1 × 10^8^ CFU/mL bacteria. As shown in Figure 1, *B. breve* (1 × 10^8^ CFU/mL) caused a modest time-dependent enhancement (25% increase) in Caco-2 TER (Figure 1A) while *B. longum* did not have any effect on Caco-2 TER (Figure 1B). In contrast, *Bifidobacterium bifidum* (BB) treatment resulted in a marked increase (50–80%) in Caco-2 TER by day 1 (Figure 1C), which was maintained throughout the experimental period. Conversely, BB caused a time-dependent decrease in apical-to-basolateral flux of paracellular marker dextran 10 kDa (Figure 1D), indicating an increase in intestinal epithelial TJ barrier function. We next examined whether the effect of *Bifidobacterium bifidum* on Caco-2 intestinal epithelial TJ barrier was species-wide or strain-specific effect. In the following studies, the effect of five different BB strains (referred to as BB1-BB5) on Caco-2 TER was determined. As shown in Figure 1E, BB1 (1 × 10^8^ CFU/mL) caused a marked increase in Caco-2 TER while BB2 and BB3 caused a modest enhancement. On the other hand, BB4 did not have a significant effect on Caco-2 TER, and BB5 caused a small decrease in Caco-2 TER (Figure 1E). Collectively, these data suggested that the BB effect on Caco-2 TJ barrier function was strain-specific. Next, to determine whether the bacteria-secreted product or live bacteria was required for the BB1 enhancement of the Caco-2 TJ barrier, the effect of BB1 bacterial supernatant or heat-killed BB1 on Caco-2 TER was examined. Neither the supernatant nor heat-killed BB1 affected the Caco-2 TER (Figure 1F), suggesting that a live bacterial-epithelial cell interaction was required for the BB1 enhancement of Caco-2 intestinal epithelial TJ barrier.

### 2.2. Involvement of Toll-like Receptors in BB1 Enhancement of Caco-2 TJ Barrier Function

**Figure 2:**
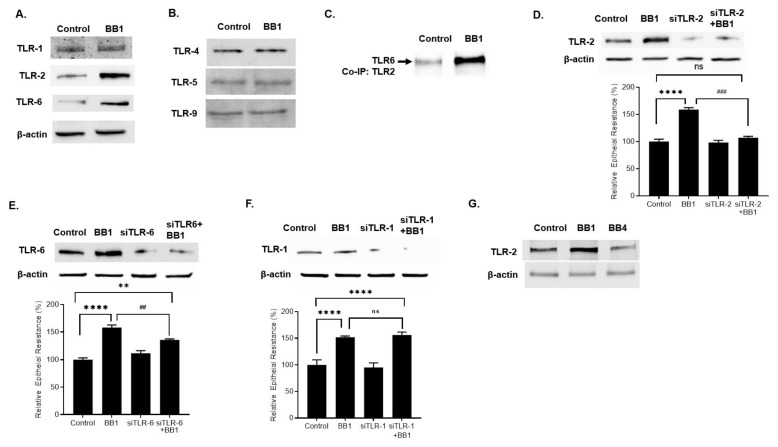
Effect of BB (1 × 10^8^ CFU/mL) on Caco-2 TLRs. (**A**) BB1 caused an increase in Caco-2 TLR-2 and TLR-6 protein expression, but not TLR-1. (**B**) BB1 did not affect the protein expression of Caco-2 TLR-4, TLR-5, or TLR-9. (**C**) BB1 caused an increase in TLR-2/TLR6 interaction in Caco-2 monolayers. Total cell extracts were prepared and subjected to co-immunoprecipitation (co-IP) as described in Materials and Methods. (**D**) TLR-2 siRNA transfection caused a near-complete depletion of TLR-2 protein expression, prevented the BB1 induced increase in TLR-2 protein expression, and prevented the BB1-induced increase in Caco-2 TER (24-h experimental period); **** *p* < 0.0001 vs. control; ### *p* < 0.001 vs. BB1. (**E**) The siRNA induced knock-down of TLR-6 caused inhibition of BB1 induced increase in Caco-2 TER. ** *p* < 0.01 vs. control; **** *p* < 0.0001 vs. control; ## *p* < 0.01 vs. BB1; ns: not significant. (**F**) Knocking-down TLR-1 by siRNA transfection did not prevent the BB1 increase in Caco-2 TER; **** *p* < 0.0001 vs. control. (**G**) BB1 but not BB4 caused an increase in TLR-2 protein expression in Caco-2 monolayers (1-day experimental period).

Toll-like receptors (TLRs) are pathogen recognition receptors and have been shown to play a critical role in bacterial/host cell interactions in intestinal epithelial cells [24]. Previous studies have shown that TLRs, including TLR-4, TLR-5, TLR-9, and TLR-2, specializes in bacterial recognition in the intestinal epithelial cells [25,26]. In the following studies, the effect of BB1 on the expression of TLR-2, TLR-4, TLR-5, and TLR-9 on filter-grown Caco-2 monolayers was examined. BB1 (1 × 10^8^ CFU/mL) caused an increase in TLR-2 protein expression by day 1 (Figure 2A). On the other hand, BB1 treatment did not affect the protein expression of TLR-4, TLR-5, or TLR-9 in Caco-2 monolayers (Figure 2B). Interestingly, BB1 also caused an increase in TLR-2 heterodimer partner TLR-6 protein expression but not TLR-1 (Figure 2A). Next, Caco-2 TLR-2 was immunoprecipitated, and co-immunoprecipitation of TLR-6 and TLR-1 was determined. As shown in Figure 2C, BB1 caused a marked increase in TLR-6 co-immunoprecipitation with TLR-2. BB1 did not cause an increase in TLR-1 co-immunoprecipitation (data not shown). Next, to determine the involvement of the specific TLR-2 receptor complex, TLR-2/TLR-1 or TLR-2/TLR-6, in BB1 enhancement of Caco-2 TER, the effect of small interference RNA (siRNA) induced knock-down of TLR-2, TLR-1, or TLR-6 on BB1 enhancement of Caco-2 TER was examined. The TLR-2 siRNA transfection caused a near-complete depletion of TLR-2 in filter-grown Caco-2 monolayers (Figure 2D). The siRNA-induced knock-down of TLR-2 inhibited the BB1-induced increase in TLR-2 protein expression (Figure 2D) and the increase in Caco-2 TER (Figure 2D). The siRNA-induced knock-down of TLR-6, but not TLR-1, inhibited the BB1-induced increase in Caco-2 TER (Figure 2E,F). Collectively, these results suggested that TLR-2/TLR-6 heterodimer but not TLR-2/TLR-1 heterodimer, played a key role in BB1 enhancement of intestinal epithelial TJ barrier function. To further investigate the strain-specific effects of BB1 on TLR-2 expression in Caco-2 monolayers, the expression of TLR-2 in response to the BB strain BB4, the strain which does not produce an enhancement in Caco-2 TJ barrier function, was examined. In contrast to BB1, BB4 did not cause an increase in Caco-2 TLR-2 expression (Figure 2G), further suggesting that the increase TLR-2 up-regulation was required for the BB enhancement of the TJ barrier.

### 2.3. Mechanism of BB1 Attachment to Caco-2 Apical Membrane Surface and the Signaling Pathway Mediating the BB Enhancement of Intestinal TJ Barrier

**Figure 3:**
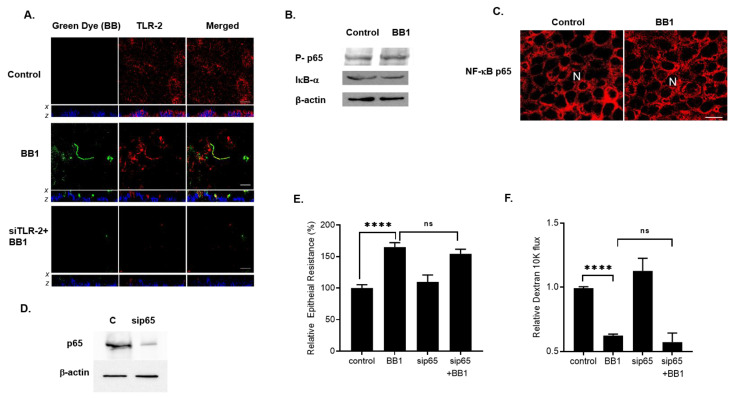
Intracellular mechanisms involved in BB1 enhancement of Caco-2 intestinal epithelial TJ barrier. (**A**) BB1 attachment to the Caco-2 apical membrane surface. In control Caco-2 monolayers, TLR-2 (red) is diffusely distributed on the apical membrane surface. LA1 (green) adheres to Caco-2 apical membrane at sites of TLR-2 (yellow in merge panel, middle row, 2-h treatment). Nuclei are shown in blue color. *x–z* stacks showed that the BB1 and TLR-2 co-localized on the apical membrane of Caco-2 cells. The siRNA knock-down of TLR-2 (bottom row) showed loss of TLR-2 staining and prevention of BB1 attachment. White bars = 10 μm. (**B**) BB1 did not affect the activation of NF-κB p65 as assessed by phosph-p65 expression or the degradation of IκB-α in Caco-2 monolayers (1-day experimental period). (**C**) BB1 did not affect the cytoplasmic-to-nuclear translocation of NF-κB p65 as determined by immunofluorescence imaging, 40×. (**D**) NF-κB p65 siRNA transfection caused a near-complete depletion of NF-κB p65 protein expression. (**E**) NF-κB p65 siRNA transfection did not prevent the BB1-induced increase in Caco-2 TER and (**F**) decrease to dextran 10 kDa flux (1-day experimental period); **** *p* < 0.001 vs. control; ns: not significant.

In the following set of experiments, we examined the intracellular process which mediated the BB1-induced enhancement of TJ barrier function in Caco-2 monolayers. We first investigated the possible involvement of TLR-2 in BB1 attachment to the Caco-2 apical membrane surface. In control Caco-2 monolayers, TLR-2 was expressed diffusely on the apical membrane surface, as shown in Figure 3A. The attachment of BB1 to Caco-2 apical membrane surface resulted in a rapid re-distribution and focal aggregation of TLR-2 at discrete points of BB1 attachments along the apical membrane surface. BB1 attachment (green) was shown to co-localize at the focal points of TLR-2 aggregation (red) on Caco-2 apical membrane surface (Figure 3A). The siRNA-induced knock-down of TLR-2 prevented the BB1 attachment to the apical membrane surface, indicating the requirement of TLR-2 on the apical membrane surface for the BB1 attachment.

**Figure 4:**
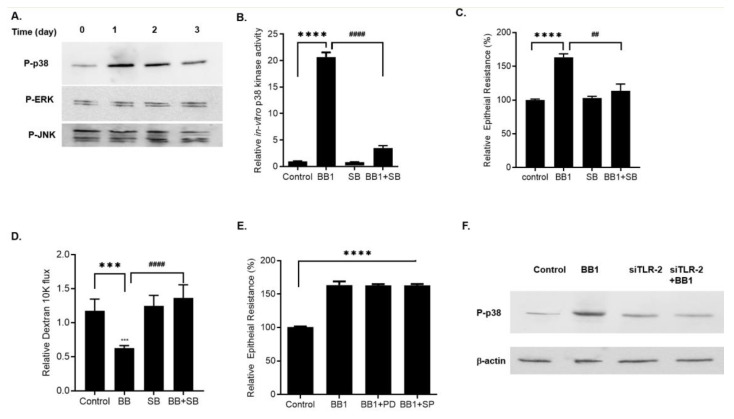
Effect of BB1 on MAP activation in filter-grown Caco-2 monolayers. (**A**) BB1 caused a time-dependent increase in phosphorylated p38 kinase but not ERK1/2 or JNK phosphorylation. (**B**) BB1 caused an increase in p38 kinase activity as determined by ELISA-based in vitro kinase activity. Pre-treatment with p38 kinase inhibitor SB-203580 (10 μM) 1 h prior to BB1 treatment (1 day) prevented the BB1–induced increase in p38 kinase activity. **** *p* < 0.0001 vs. control, #### *p* < 0.0001 vs. BB1. (**C**) SB-203580 prevented the BB1–induced increase in Caco-2 TER. **** *p* < 0.0001 vs. control, ## *p* < 0.01 vs BB1; and (**D**) decrease in dextran 10 kDa flux (1-day experimental period). *** *p* < 0.001 vs. control, #### *p* < 0.001 vs. BB1. (**E**) Pre-treatment with ERK1/2 inhibitor, PD 98059 (100 μM) or JNK inhibitor SP600125 (10 μM) did not prevent the BB1-induced increase in Caco-2 TER. **** *p* < 0.0001 vs. control. (**F**) TLR-2 siRNA transfection prevented the BB1-induced p38 kinase phosphorylation in Caco-2 monolayers.

Next, we examined the intracellular signaling pathways mediating the BB1/TLR-2 receptor complex-regulated increase in Caco-2 TJ barrier. We specifically examined the involvement of NF-κB and MAP kinase pathways, the two pathways known to regulate TJ barrier function [2,27,28,29,30,31]. BB1 addition to Caco-2 apical membrane did not cause activation of NF-κB p65 as measured by phospho-NF-κB p65 expression, IκB-α degradation, and NF-κB p65 nuclear translocation (Figure 3B,C). To further assess the possible involvement of the NF-κB pathway in BB1 enhancement of the Caco-2 intestinal epithelial TJ barrier, NF-κB p65 was selectively knocked-down by siRNA transfection (Figure 3D). The silencing of NF-κB p65 did not affect the BB1-induced increase in Caco-2 TER or the decrease in dextran permeation (Figure 3E,F), suggesting that the BB1 enhancement of the Caco-2 TJ barrier function was not dependent on the NF-κB pathway. Next, we investigated the possible involvement of MAP kinase pathways in BB1 enhancement of Caco-2 TJ barrier function. The effect of BB1 (1 × 10^8^ CFU/mL) on ERK1/2, p38 kinase, or JNK activation was determined by assessing ERK1/2, p38 kinase, and JNK phosphorylation in Caco-2 monolayers. BB1 caused an increase in phosphorylation of p38 kinase in Caco-2 cells, starting at day 1 and continuing up to day 3, as determined by p-p38 kinase immunoblotting (Figure 4A). In contrast, BB1 did not cause an increase in ERK1/2 or JNK phosphorylation (Figure 4A). BB1 effect on Caco-2 p38 kinase activation was also confirmed by an ELISA-based in vitro kinase assay, using ATF-2 as the substrate. BB1 caused a marked increase in in-vitro kinase activity of Caco-2 p38 kinase, and inhibition of p38 kinase by a pharmacologic inhibitor, SB 203580 (10 μM) inhibited BB1–induced activation of p38 kinase (Figure 4B).To confirm the requirement of p38 kinase activation on BB1-induced enhancement of Caco-2 TJ barrier, the effect of p38 kinase inhibitor was determined. The inhibition of p38 kinase by pharmacologic inhibitor SB 203580 prevented the BB1 increase in Caco-2 TER (Figure 4C) and decreased in dextran-10kd permeation (Figure 4D). On the other hand, ERK1/2 inhibitor, PD 98059 (100 μM), or JNK inhibitor, SP 600125 (10 μM) did not have any effect on the BB1–induced enhancement of Caco-2 TJ barrier (Figure 4E). Next, to examine the regulatory involvement of TLR-2 receptor complex on p38 kinase activation, we knocked-down TLR-2 expression by siRNA transfection in Caco-2 monolayers and examined the effect of BB1 on p38 kinase activation. Knocking-down TLR-2 prevented the BB1 induced activation and phosphorylation of p38 kinase (Figure 4F), indicating that BB1-activation of TLR-2 pathway was required for the BB1-activation of p38 kinase and the subsequent enhancement of intestinal epithelial TJ barrier.

### 2.4. Effect of BB1 on Mouse Intestinal Permeability In-Vivo and Protection against DSS-Induced Increase in Colonic Permeability and Colitis

**Figure 5:**
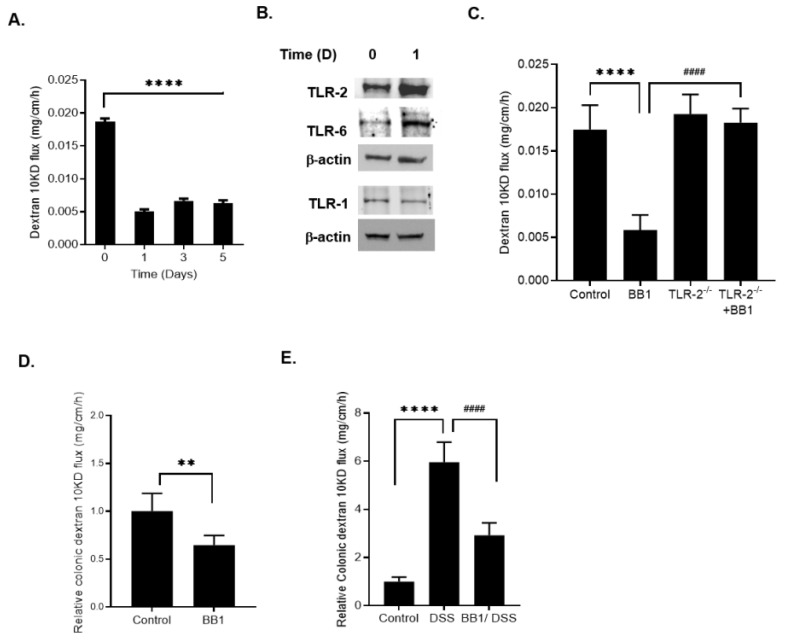
Effect of BB1 on mouse intestinal permeability in-vivo and DSS-induced increase in mouse colonic permeability and inflammation. (**A**) BB1 (1 × 10^9^ CFU/mL) caused a time-dependent decrease in mouse intestinal permeability to dextran-10 kDa; **** *p* < 0.0001 vs. control. (**B**) BB1 oral gavage caused an increase in TLR-2 and TLR-6 but not TLR-1 protein expression in mouse small intestinal tissue. (**C**) BB1 induced enhancement of mouse intestinal permeability was inhibited in TLR-2^−/−^ mice. **** *p* < 0.0001 vs. control; #### *p* < 0.0001 vs. BB1. (**D**) BB1 administration caused a decrease in mouse colonic permeability to dextran-10 kDa; ** *p* < 0.01 vs. control. (**E**) DSS (3% *w*/*v*) caused a marked increase in mouse colonic permeability to dextran-10 kDa, and BB1 prevented the DSS-induced increase in mouse colonic permeability over the 7-day experimental period. (BB1 treatment started 2 days prior to the 7-day DSS treatment). **** *p* < 0.0001 vs. control; #### *p* < 0.0001 vs. DSS treatment.

In the following studies, the effect of BB1 on mouse intestinal epithelial barrier function was examined in-vivo. The effects of BB1 administration via oral-gastric gavage on mouse intestinal permeability was determined in-vivo by recycling perfusion of the small intestinal segment in live mice as previously described [2,25,32]. BB1 (1 × 10^9^ CFU/mL) administration caused an enhancement in the intestinal TJ barrier as evidenced by the decrease in mouse small intestinal permeability to paracellular marker dextran-10 kDa (Figure 5A). By day 1, there was a marked decrease in mouse intestinal permeability, which persisted up to the 5 days of the experimental period (Figure 5A). Next, the effect of BB1 on mouse intestinal tissue expression of TLR-2 was determined. BB1 oral administration caused an increase in intestinal tissue TLR-2 by day 1 (Figure 5B). The BB1 administration also caused an increase in intestinal tissue TLR-6, but not TLR-1, protein expression (Figure 5B), consistent with the above results with the filter-grown Caco-2 monolayers. To determine the requirement of TLR-2 in BB1 induced decrease in mouse small intestinal permeability, the effect of BB1 on TLR-2 deficient (TLR-2^−/−^) mice was determined. BB1 decrease in mouse small intestinal permeability was significantly inhibited in TLR-2^−/−^ mice (Figure 5C). Next, the possible protective effect of BB1 against DSS-induced increase in colonic permeability and colitis was determined. We recently showed that DSS-induced increase in colonic permeability (by day 1) precedes the development of colitis, which occurs by day 4–5 [1]. The oral administration of 3% DSS for 7 days caused a time-dependent increase in colonic permeability, starting day 1, and development of mild colitis by day 4, and more severe colitis by day 7 [1]. Herein, we found that BB1 (1 × 10^9^ CFU/mL) administration caused a rapid decrease in colonic permeability (Figure 5D). The daily BB1 treatment starting 2 days prior to DSS administration and continuing throughout the entire 7 days of DSS administration inhibited the DSS-induced increase in colonic permeability (Figure 5E).

**Figure 6:**
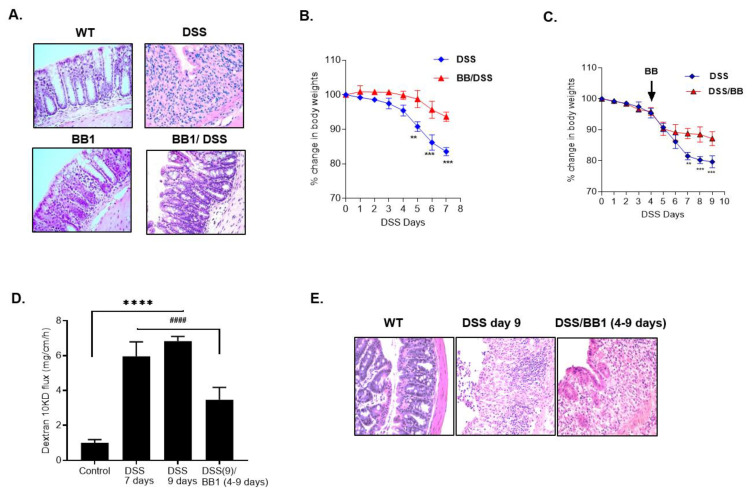
Protective and therapeutic effects of BB1 on DSS-induced colitis. (**A**) H & E staining of colonic mucosa after DSS administration showed ulceration of colonic mucosal surface with loss of surface epithelium and crypts and diffuse infiltration of inflammatory cells. BB1 administration attenuated the DSS-induced ulceration and loss of colonic mucosal architecture and the inflammation was reduced (BB1 treatment started 2 days prior to the 7-day DSS treatment). H & E, 200×. (**B**) BB1 prevented the DSS-induced body weight loss. ** *p* < 0.01 vs. DSS; *** *p* < 0.001 vs. DSS. (**C**) BB1 treatment attenuated the DSS-induced body weight loss over the 9-day experimental period. (BB1 treatment started 4 days after DSS administration and continued up to the 9-day experimental period). ** *p* < 0.01 vs. DSS; *** *p* < 0.001 vs. DSS. (**D**) DSS caused a marked increase in mouse colonic permeability to dextran-10 kDa over 9 days of DSS administration, and BB1 promoted the healing from the DSS-induced increase in mouse colonic permeability over the 9-day experimental period. (BB1 administration started 4 days after DSS treatment and continued up to the 9-day experimental period) **** *p* < 0.0001 vs. control; #### *p* < 0.0001 vs. DSS. (**E**) H & E staining of colonic mucosa after DSS administration (9-day experimental period) showed ulceration of colonic mucosal surface with loss of surface epithelium and crypts and diffuse infiltration of inflammatory cells. BB1 treatment 4 days after DSS administration attenuated the DSS-induced inflammation (BB1 treatment started 4 days after DSS treatment and continued up to 9 days). H & E, 200×.

The BB1 administration also attenuated the development of colitis (Figure 6A) and weight loss in the BB1-treated mice (Figure 6B). We also examined the therapeutic effects of BB1 on DSS-induced colitis by administration of BB1 starting day 4, after the onset of colitis. The BB1 treatment, starting on day 4 of DSS administration and continuing to day 9 of DSS administration, prevented further weight loss, reversed the DSS-induced increase in colonic permeability, and promoted mucosal healing (Figure 6C–E). These data also suggested that BB1 promotes the restoration of the colonic barrier and healing of the DSS-induced colitis.

## 3. Discussion

There is growing scientific evidence supporting the integral role of commensal and probiotic bacteria in maintaining and promoting intestinal homeostasis and intestinal epithelial barrier function. Recent studies also suggest that therapeutic targeting of the intestinal TJ barrier could be important in the management of gastrointestinal disorders associated with defective intestinal TJ barrier function [33,34,35,36]. Among the various probiotic bacteria reported to date, *Bifidobacterium* spp., is one of the most widely utilized probiotic bacteria. *Bifidobacterium* spp. are the first to colonize the human intestine, a phenomenon driven by the bifidogenic activities of certain mother milk-derived oligosaccharides [16,37]. Consequently, they account for nearly 80% of microorganisms in the intestinal tract of breastfed infants. *B. breve, B. bifidum, B. longum,* and *B. infantis* are the commonly detected bacteria at the infant stage, with *B. bifidum* being the most prominent species, followed by *B. breve, B. longum,* and *B. infantis* [15,17,38]. Bifidobacteria have been commercially exploited as probiotic agents due to their well-established health benefits and GRAS (Generally Recognized As Safe) status [39]. The health-promoting biological activities exerted by Bifidobacteria are numerous and include the establishment of a healthy microbiota in preterm infants [40], protection against pathogens [41], promoting an anti-inflammatory environment through modulation of host immune response [42], production of vitamins and short chain fatty acids, and enhancement of intestinal gut barrier [9,10]. Being an innate and integral member of the human gut, *Bifidobacterium* has been proven to be essential for maintaining intestinal epithelial barrier integrity [18]. Indeed, a recent study classified the microbiota of ulcerative colitis (UC) patients by 16SrRNA microbial profiling revealed a substantial decrease of bifidobacteria, notably *B. bifidum*, suggesting that this probiotic genus could play a biological role in the etiology of UC and also highlighted the importance of *B. bifidum* as a potential microbial biomarker for UC [19]. The reports of the beneficial effect of *Bifidobacteria* in treating various GI disorders have been conflicting. For example, *B. bifidum*, has been shown to improve intestinal integrity in a rat model of necrotizing enterocolitis (NEC) [43]. On the other hand, recent studies indicated that *Bifidobacterium breve* BBG-001, had neither effects on intestinal barrier function nor clinical efficacy to prevent NEC [44].

There exists an important gap in the scientific knowledge as to which *Bifidobacterium* species are responsible for the enhancement of the intestinal TJ barrier and which species have the maximal effect. Additionally, it remains unclear whether the *Bifidobacterium*-based therapies can restore the intestinal TJ barrier. The primary goal of this study was to identify the specific *Bifidobacterium* species that can induce maximal enhancement of intestinal TJ barrier function. The secondary aim was to determine whether the *Bifidobacterium* bacterial targeting of the intestinal TJ barrier can protect against the development of colitis in an animal model of colitis.

In our studies, we screened three of the most common *Bifidobacterium* species, *B. breve; B. longum, and B. bifidum* that are most present in the intestinal tract. We found that *B. breve* and *B. longum* had modest (~35% increase in Caco-2 TER) or no effect on Caco-2 TJ barrier function. In contrast, *B. bifidum* (BB) produced a rapid, sustained, and marked enhancement (70–80% increase in TER) in intestinal epithelial TJ barrier function, starting from day 1 of BB colonization. Our studies also revealed that only one particular BB strain, BB1, caused the maximal enhancement in the intestinal TJ barrier, while other BB strains had modest (BB2 and BB3) or no effect (BB4 and BB5), indicating a strain-specific effect of BB on intestinal epithelial TJ barrier function. These findings suggested that BB1 has the unique biological characteristic to produce the maximal enhancement in the intestinal TJ barrier and that the TJ barrier enhancing effect was strain-specific effect. Previously published studies with BB also showed that the BB strains studied had either minimal or modest effect on the intestinal TJ barrier [20,45]. These studies found that the various BB strains studied caused an increase in Caco-2 TER, ranging from 5–34% increase in TER [20]. In the studies described herein, we found a unique BB stain, BB1, which induced about 70–80% increase in Caco-2 TER and over 70% decrease in mouse intestinal permeability to macromolecular (dextran 10 kDa) flux.

Intestinal epithelial cells are uniquely positioned in the gastrointestinal tracts to recognize microbial components through various pattern recognition receptors (PRRs), including Toll-like receptors (TLRs) [24,30,46]. The enterocyte membrane surface expresses TLR-2, TLR-4, TLR-5, and TLR-9, which are known to play a pivotal role in sensing and responding to commensal and pathogenic microbes in-vivo [47,48,49]. Our results herein, combined with our previous publications, show that TLR-2 is present only on the apical membrane surface of intestinal epithelial cells while TLR-4 and TLR-5 are present on the basolateral membrane surface under normal quiescent conditions [1,50]. In our studies, BB1 addition to the apical membrane surface caused a rapid increase in TLR-2 expression in Caco-2 monolayers and TLR-2-dependent enhancement in Caco-2 TER. In the absence of TLR-2, BB1 is not able to induce an increase in Caco-2 TJ barrier function, confirming that the BB1 enhancement of Caco-2 TJ barrier function is mediated by TLR-2 signal transduction pathway. Similarly, in a mouse model, the BB1 enhancement of the mouse intestinal epithelial barrier is also accompanied by an increase in intestinal tissue TLR-2 expression, and the BB1 enhancement of intestinal TJ barrier is inhibited in TLR-2 deficient mice.

TLR-2 forms a heterodimer complex with either TLR-6 or TLR-1 subunits and recognizes the diacylated or triacylated lipoproteins/lipopeptides present on Gram positive or Gram negative bacteria, respectively [36,46,51]. Our studies show that BB1 causes an increase in the expression of TLR-6, but not TLR-1, correlating with the increase in TLR-2 expression. Consistent with the prior studies showing that Gram positive bacteria preferentially interact with TLR-2/TLR-6 dimer, in our studies, the BB1 up-regulation of the intestinal epithelial TJ barrier was mediated by the TLR-2/TLR-6, but not TLR-2/TLR-1 heterodimer.

Our data also suggested that the BB1 enhancement of Caco-2 TJ barrier required direct live bacterial-epithelial cell interaction, as heat-killed BB1 or BB1 bacterial supernatant did not affect the TJ barrier. The BB1/Caco-2 epithelial cell-binding studies provide a novel insight into the role TLR-2 plays in BB1 and intestinal epithelial cells interaction. The BB1 produced a rapid redistribution of the apical membrane surface TLR-2 localization and attached only at the points of focal aggregation of TLR-2. The BB1 was not able to attach to Caco-2 apical membrane surface in the absence of TLR-2 and did not cause an enhancement in Caco-2 TJ barrier function. Thus, these findings show for the first time that TLR-2 plays a critical role in BB/intestinal epithelial cell attachment and subsequent up-regulation of the Caco-2 TJ barrier function. The Bifidobacterium/TLR-2 interaction has been shown previously in dendritic cells (DC) to exert immunoinhibitory effects and regulate the production of anti-inflammatory cytokine IL-10 [52]. *Bifidobacteria* have also been shown to inhibit the production of TNF-α and IL-6 via interaction with TLR2 [53]. In these studies, the authors found that the interaction of lactic acid bacteria, including *Bifidobacterium*, in the gut immune system was regulated through the interaction of lipoprotein with TLR-2. Bifidobacterium was shown to induce immunoinhibitory effects in a TLR-2 dependent manner and nucleotide-binding oligomerization domain-2 (NOD-2)-independent manner [53].

In this study, we also investigated the signaling pathways involved in the TLR-2-dependent enhancement effect on intestinal TJ barrier function. The TLR-2 signal transduction pathway is known to activate NF-κB and MAP Kinases, ERK1/2, JNK, and p38 kinase [54,55,56,57,58]. Kausahal et al. demonstrated that *Bifidobacterium bifidum* improved the phagocytic potential of macrophages in mice by activating NF-κB signaling pathway and inducing cytokine production [59]. Other investigators have shown that various probiotic bacteria caused an increase in TLR-2 expression in mice intestinal dendritic cells (DCs) and macrophages, and in human myeloid DCs [60,61,62]. It has also been shown that supernatant from *Bifidobacterium* species causes maturation, activation, and survival of DCs via activation of p38 kinase [63]. Together, these studies demonstrate that probiotic bacteria exert their immune-modulatory effect through activation of TLR-2 signal-transduction pathway. On the other hand, earlier studies have also shown that *Bifidobacterium* activation of TLR-2 plays an important role in maintaining intestinal epithelial TJ barrier function. Stimulation with the synthetic TLR-2 agonist PCSK efficiently preserves TJ-associated barrier integrity [64,65]. Our studies herein show that the BB1 did not cause NF-κB activation or that NF-κB was involved in the BB1 up-regulation of intestinal epithelial TJ barrier. The BB1 enhancement of the intestinal epithelial TJ barrier was regulated by the activation of p38 kinase pathway, and the BB1 induced activation of p38 kinase was inhibited in the absence of TLR-2, suggesting that the TLR-2 receptor complex induced enhancement of the TJ barrier was mediated by the p38 kinase activation. We also examined the possibility that BB1 induced enhancement of TJ barrier function might be associated with changes in TJ protein expression. BB1 effect on TJ proteins including occludin, claudin-1, claudin-2, claudin-3, and ZO-1 was examined. In these preliminary studies, BB1 treatment did not have a significant effect on protein expression of ZO-1, claudin-1, caludin-2, or claudin-3, but caused an increase in occludin expression in Caco-2 monolayers (data not shown), suggesting the possibility that the increase in occludin expression could, in part, contribute to the BB1 enhancement of intestinal TJ barrier function.

Our proof-of-concept animal studies herein also extend on the Caco-2 epithelial TJ regulation studies to demonstrate the therapeutic efficacy of probiotic bacterial targeting of the intestinal TJ barrier to prevent or treat colitis in an animal model. Our studies showed that BB1 induced a rapid and marked enhancement in the mouse intestinal epithelial barrier in a TLR-2-dependent manner. The DSS-induced colitis is dependent on the loss of intestinal epithelial barrier function and the entry of luminal bacteria and their products into the lamina propria, contributing to the inflammatory response [66,67,68]. The oral administration of 3% DSS causes an early increase in mouse intestinal permeability, which precedes the development of mild histologic inflammation by day 4 and more severe colitis by day 7 [1,69]. Our data show that BB1 causes an increase in mouse intestinal barrier function in a TLR-2-dependent manner and prevents the DSS-induced increase in colonic permeability and the subsequent development of colitis. Our studies also show that BB1 treatment following the onset of DSS colitis is also effective in treating and healing the colonic barrier and colitis. Thus our studies show that the BB1 targeting of the intestinal TJ barrier function is effective in both prevention and treatment of DSS-induced colitis.

In conclusion, our results show for the first time that *Bifidobacterium bifidum* strain BB1 has the unique bacterial characteristic of inducing a rapid and sustained enhancement in intestinal TJ barrier function in a TLR-2 and p38 kinase-dependent manner and protects against the DSS-induced colitis by preserving the intestinal TJ barrier in a TLR-2-dependent manner. A proof-of-concept clinical studies to assess the BB1 effect on the enhancement of intestinal epithelial barrier in patients with intestinal permeability disorders, including Crohn’s disease and ulcerative colitis, are in progress.

## 4. Materials and Methods

### 4.1. Reagents

DMEM, trypsin, FBS, glutamine, penicillin, streptomycin, PBS, and horseradish peroxidase (HRP)-conjugated secondary antibodies for Western blot analysis were purchased from Invitrogen Life Technologies (San Francisco, CA, USA). TLR-2 (ab68159).

TLR-2 (ab191458); TLR-1(ab180798); TLR-6 (ab37072) antibodies were purchased from Abcam (Cambridge, MA, USA). Phospho-NF-κB p65 (sc-136548), IκB-α (sc-1643), NF-κB p65 (sc-8008), and β-actin (sc-47778) antibodies were purchased from Santa Cruz (Dallas, TX, USA). Phospho-p38 kinase (9211) antibody; phospho-ERK1/2 (9101) antibody and phospho-JNK (9255) antibody were purchased from Cell Signaling (Boston, MA, USA). All other chemicals were of reagent grade and were purchased from Sigma-Aldrich (St. Louis, MO, USA), VWR (Aurora, CO, USA), or Fisher Scientific (Pittsburgh, PA, USA).

### 4.2. Determination of Epithelial Monolayer Resistance and Paracellular Permeability

The transepithelial electrical resistance (TER) of the filter-grown Caco-2 intestinal monolayers was measured using an epithelial voltohmeter (EVOM; World Precision Instruments, Sarasota, FL, USA), as previously reported [1,70]. Electrical resistance was measured in 5% difference on 3 consecutive measurements. Caco-2 paracellular permeability was assessed by measuring the luminal-to-serosal flux rate of a paracellular probe, fluorescein isothiocyanate-labeled FITC dextran 10 kDa (mol wt.: 10,000 g/mol). For determination of mucosal-to-serosal flux rates, a known concentration (25 μg/mL) of FITC dextran 10 kDa was added to the apical solution at the beginning of each experiment. After each experimental period, the solution from the basolateral chamber was collected and measured in a fluorescence microplate reader (Biotek Flx 800). All flux studies were carried out at 37 °C and filter-grown Caco-2-monolayers having epithelial resistance of 400−550 Ω·cm^2^ were used. All of the permeability experiments were repeated 3 to 4 times in triplicate.

### 4.3. Preparation of Bacterial Culture and Cell-Free Culture Supernatant

Bifidobacterium species and strains were purchased from ATCC: B. bifidum, B. breve, and B. longum. These bacteria were grown overnight in MRS broth (Difco, Detroit, MI, USA) at 37 °C in anaerobic jars with shaking. Live bacteria were spun down by centrifuging at 12,000× *g* for 10 min and pellets were stored at −80 °C for further experiments. Supernatants were separated from spun down bacteria, filtered, and diluted in cell culture media DMEM (with no FBS and no antibiotics) for further use. Viable Bifidobacterium were heat-killed by incubations in a water bath at 60 °C for 1 h. The viable count was performed to make sure no viable bacteria survived. Bacteria were stored at X80 °C as heat-killed Bifidobacterium. For treating Caco-2 monolayers, the bacterial pellet was suspended in DMEM and diluted to OD600nm = 0.135 (1 × 10^8^ colony-forming units (CFU)) in the same media and applied to the apical surface of cell monolayers.

### 4.4. Determination of Mouse Small Intestinal Permeability In-Vivo and Measurement of Trans-Epithelial Electrical Resistance

Studies were approved by the Penn State University and University of New Mexico Institutional Animal Care and Use Committee. TLR-2 null (TLR-2−/−) and wild-type (WT) mice (both of C57BL/6 background) of 9–12 weeks of age were obtained from The Jackson Laboratory (Bar Harbor, ME, USA). BB effect on intestinal permeability in an in-vivo mouse model system was determined using a recycling intestinal perfusion method [1]. For in vivo studies, 1 × 10^9^ CFU of BB in 200 μL PBS were administered daily by oral-gastric gavage and mouse intestinal permeability was measured at different treatment periods. A 6–8 cm segment of mouse small intestine was isolated and cannulated with a small diameter plastic tube (in an anesthetized mouse) and continuously perfused with 5 mL Krebs-phosphate saline buffer for a 2 h perfusion period. An external recirculating pump was used to recirculate the perfusate at a constant flow rate (0.75 mL/min). The intestinal permeability was assessed by measuring luminal-to-serosal flux rate of paracellular probe, Texas Red-labeled dextran (MW = 10,000 g/mol) [27,71].

### 4.5. Induction of DSS-Colitis and Determination of Mouse Colonic Permeability In Vivo

Mice received 3% (*w*/*v*) DSS (molecular weight 36,000–50,000, MP Biomedicals, Santa Ana, CA, USA) in autoclaved drinking water for 7 days. The body weights of mice were monitored daily, and histological grading of colitis lesions were performed as described previously [69,72]. To quantify the extent of mucosal damage, a segment from the distal colon was fixed in 4% paraformaldehyde, paraffin embedded, sectioned (5 μm), and stained with hematoxylin and eosin (H & E). For prevention studies, BB1 was gavaged starting 2 days prior to DSS treatments and continued throughout DSS administration. The 2 days of pre-treatment with BB1 were chosen based on the positive effect of BB1 on our data showing that BB1 enhancement of intestinal TJ barrier occurs by day 1, and this ensures that the barrier enhancing effects were present by the time DSS was administered. For active treatment studies, BB1 was gavaged starting at day 4 post DSS administration and continued up to 9 days. The colonic permeability was measured by recycling colonic perfusion method, as previously described at the end of 7 or 9 days of DSS treatment [1]. The colonic permeability was assessed by measuring the luminal-to-serosal flux rate of a paracellular probe, Texas Red-labeled dextran (mol wt.: 10,000 g/mol).

### 4.6. Assessment of Protein Expression by Western Blot Analysis

Protein expression from Caco-2 cells was assessed by Western blot analysis, as previously described [73]. Cells were lysed with lysis buffer on ice for 30 min. Cell lysates containing 10–20 µg of protein were separated on an SDS-PAGE gel. Proteins from the gel were transferred to a nitrocellulose membrane overnight. The membrane was incubated for 2 h in blocking solution (5% dry milk in TBS-Tween-20 buffer) and then incubated with antibody in blocking solution. After a wash in TBS-1% Tween buffer, the membrane was incubated in secondary antibody and developed using enhanced chemiluminescence reagents on ChemiDoc Gel Imaging (Biorad, Hercules, CA, USA). For immunoprecipitation experiments, cell lysates were pre-cleared with, and then protein concentration was determined. Cell lysates (50 μg) were incubated with 10 μg of TLR-2 antibody for 2 h at 4 °C followed by the addition of 50 μL Dynabeads (Thermofisher, Waltham, MA, USA). After 1 h at room temperature, the beads were washed 3 times with lysis buffer. The protein bound beads were boiled for 7 min in 2× sample buffer and subjected to Western blot analysis.

### 4.7. Immunostaining of NF-κB p65

Cellular localization of the transcription factor NF-κB p65 was assessed by an immunofluorescent Ab-labeling technique as previously described [28,29]. At the end of the experimental period, filter-grown Caco-2 monolayers were washed twice in cold PBS and were fixed with methanol for 20 min. Then, cells were permeabilized with 0.1% Triton X-100 in PBS at room temperature for 20 min. The Caco-2 monolayers were then incubated in a blocking solution composed of BSA and normal donkey serum in PBS for 1 h. Cells were then labeled with primary Abs in blocking solution overnight at 4 °C. After being washed with PBS, the filters were incubated in FITC-conjugated secondary Ab for 1 h at room temperature. Prolong Gold antifade reagent with DAPI was used to mount the filters onto the coverslips. Immunostaining of NF-κB p65 was visualized, and images obtained using a Nikon fluorescence microscope equipped with Axiocam digital camera in automatic mode. Images were processed with Zen software (Zeiss).

### 4.8. Confocal Immunofluorescence

Immuno-localization of BB1 and TLR-2 was assessed by confocal immunofluorescence. BB1 was labeled with Vybrant DiO cell-labeling solution (Thermo Fisher, Waltham, MA, USA) prior to treatment. The labeled BB1 was added to Caco-2 at the apical bathing buffer solution for 2 h; monolayers were then rinsed twice in cold PBS, fixed with methanol for 10 min. The cell monolayers were then blocked in normal serum and labeled with TLR-2 primary antibody (Abcam; 191458) in blocking solution overnight at 4 °C. After being washed with PBS, the cells were incubated in Cy3-conjugated secondary antibody (Jackson ImmunoResearch Laboratories). All the primary and secondary antibodies were used at the concentrations suggested by the manufacturers. ProLong Gold antifade reagent (Invitrogen), containing DAPI as a nuclear stain, was used to mount the cell filters on glass slides. The slides were examined using a confocal fluorescence microscope (Leica SP8). Images were processed with LAS X software (Leica Microsystems).

### 4.9. Transfection of Targeted siRNA

Targeted siRNAs were obtained from Dharmacon (Chicago, IL, USA). Caco-2 monolayers were transiently transfected using DharmaFect transfection reagent. Briefly, 5 × 10^5^ cells per filter were seeded into a 12-well transwell plate and grown to confluence. Caco-2 monolayers were then washed twice with PBS, and 0.5 mL of Accell Media was added to the apical compartment of each filter, and 1.5 mL was added to the basolateral compartment of each filter. Five nanograms of the siRNA and 2 µL of DharmaFect reagent were added to the apical media. Non-target (NT) siRNA was used as a control. The BB experiments were carried out 72 h after transfection. The efficiency of silencing was confirmed by Western blot analysis.

### 4.10. ELISA-Based In Vitro p38 Kinase Activity

The p38 kinase activity was carried out according to the manufacturer’s protocol from ActiveMotif, using ATF-2 as the substrate [73]. The plates were washed 3 times with PBS, incubated with blocking solution (1 mg/mL BSA in PBS) at 37 °C for 1 h, and then washed 3 times with PBS. The kinase reaction buffer (90 μL) (20 mM Tris-HCl [pH 7.5], 10 mM MgCl2, 50 mM NaCl, 1 mM DTT, 1 mM NaF, 50 μM ATP) provided by the manufacturer and the samples containing BB1–activated p38 kinase (10 μL) were added to each well, and the kinase reaction (phosphorylation of ATF-2) was carried out at 37 °C for 30–60 min. The reaction was stopped by removing the reaction mixtures and washing the plates 3 times with washing buffer (20 mM Tris-HCl [pH 7.4], 0.5 M NaCl, and 0.05% Tween 20). The washed plates were incubated with the anti–phospho-ATF-2 Ab (10 ng/mL) at room temperature for 1 h. Plates were washed 4 times with washing buffer, and goat anti-rabbit IgG Ab (diluted at 1:2000 in washing buffer) was added to the wells, and the plates were incubated at 37 °C for 1 h. The plates were then washed 4 times and incubated with 100 μL substrate solution tetramethylbenzidine at 37 °C for 5–15 min. A stop solution containing 0.5 N H2SO4 (100 μL) was added to stop the reaction. The absorbance at 450 nm was determined using the SpectrraMax 190 (Molecular Devices, Sunnyvale, CA, USA).

### 4.11. Statistical Analysis

Statistical significance of differences between mean values was assessed with Student t tests for unpaired data and ANOVA analysis whenever required. The values of experimental data were expressed as the mean ± S.E., and analyzed using Graph Pad Prizm 6.00 for Windows (GraphPad Software, San Diego, CA). All experiments were repeated at least 3 times to ensure reproducibility. p-values below 0.05 were considered significant.

## Data Availability

Data presented int his study is contained within the article.
